# Association Between Processed Electroencephalogram-Based Objectively Measured Depth of Sedation and Cerebrovascular Response: A Systematic Scoping Overview of the Human and Animal Literature

**DOI:** 10.3389/fneur.2021.692207

**Published:** 2021-08-16

**Authors:** Logan Froese, Joshua Dian, Alwyn Gomez, Carleen Batson, Amanjyot Singh Sainbhi, Frederick A. Zeiler

**Affiliations:** ^1^Biomedical Engineering, Faculty of Engineering, University of Manitoba, Winnipeg, MB, Canada; ^2^Section of Neurosurgery, Department of Surgery, Rady Faculty of Health Sciences, University of Manitoba, Winnipeg, MB, Canada; ^3^Department of Human Anatomy and Cell Science, Rady Faculty of Health Sciences, University of Manitoba, Winnipeg, MB, Canada; ^4^Centre on Aging, University of Manitoba, Winnipeg, MB, Canada; ^5^Division of Anaesthesia, Department of Medicine, Addenbrooke's Hospital, University of Cambridge, Cambridge, United Kingdom

**Keywords:** bispectral index, cerebrovascular autoregulation, cerebrovascular response, depth of sedation, entropy index

## Abstract

**Background:** Current understanding of the impact that sedative agents have on neurovascular coupling, cerebral blood flow (CBF) and cerebrovascular response remains uncertain. One confounding factor regarding the impact of sedative agents is the depth of sedation, which is often determined at the bedside using clinical examination scoring systems. Such systems do not objectively account for sedation depth at the neurovascular level. As the depth of sedation can impact CBF and cerebral metabolism, the need for objective assessments of sedation depth is key. This is particularly the case in traumatic brain injury (TBI), where emerging literature suggests that cerebrovascular dysfunction dominates the burden of physiological dysfunction. Processed electroencephalogram (EEG) entropy measures are one possible solution to objectively quantify depth of sedation. Such measures are widely employed within anesthesia and are easy to employ at the bedside. However, the association between such EEG measures and cerebrovascular response remains unclear. Thus, to improve our understanding of the relationship between objectively measured depth of sedation and cerebrovascular response, we performed a scoping review of the literature.

**Methods:** A systematically conduced scoping review of the existing literature on objectively measured sedation depth and CBF/cerebrovascular response was performed, search multiple databases from inception to November 2020. All available literature was reviewed to assess the association between objective sedation depth [as measured through processed electroencephalogram (EEG)] and CBF/cerebral autoregulation.

**Results:** A total of 13 articles, 12 on adult humans and 1 on animal models, were identified. Initiation of sedation was found to decrease processed EEG entropy and CBF/cerebrovascular response measures. However, after this initial drop in values there is a wide range of responses in CBF seen. There were limited statistically reproduceable associations between processed EEG and CBF/cerebrovascular response. The literature body remains heterogeneous in both pathological states studied and sedative agent utilized, limiting the strength of conclusions that can be made.

**Conclusions:** Conclusions about sedation depth, neurovascular coupling, CBF, and cerebrovascular response are limited. Much further work is required to outline the impact of sedation on neurovascular coupling.

## Introduction

The near ubiquitous use of sedation throughout a variety of critical care illnesses and its ability to help mediate the cascading secondary injury pathways in the setting of acute neurological injuries (1), highlights sedation as an important aspect of patient care in the intensive care unit (ICU). Despite the widespread use of sedation, the correlation between objectively measured depth of sedation, neurovascular coupling and cerebrovascular response/cerebral blood flow (CBF) is limited (2–5). To date, most assessments of sedation depth in the ICU occur using bedside clinical examination scoring systems, which are confounded by inter- and intra-assessor heterogeneity (6–8). In addition, such clinical exam scores do not objectively measure sedation depth at the neurological level but merely utilize the patient's response to stimuli as a surrogate for sedation depth. Moreover, there is still a major concern with overuse of sedatives as emerging evidence demonstrates an association between sedative dosing exposure and worse overall 6 month outcomes (9–12).

In specific critical illnesses, the impact that sedation has on cerebrovascular response is of paramount interest. Such is the case in the treatment of moderate/severe traumatic brain injuries (TBI), where sedation is used for its ability to reduce cerebral metabolic activity and conserve CBF with the hopes that it will maintain healthy homeostasis and reduce secondary injury (13, 14). However, recent comprehensive reviews evaluating the impact of various commonly utilized sedative agents in TBI care, and their corresponding impact on CBF/cerebrovascular response, have demonstrated conflicting results (3, 4, 15). Studies identified in these reviews failed to record objectively measured sedation depth, and only commented on the sedative agent type and dosing. Similarly, two recent works evaluating continuously measured cerebrovascular reactivity in TBI patients, in response to fluctuations in sedative agent doses, found that sedative dose change resulted in little-to-no impact on cerebrovascular reactivity (5, 16). However, again, no objective measures of sedation depth were utilized in these works. Thus, it remains unknown if there is an optimal depth of sedation in each individual patient which would promote recovery while preserving neurovascular coupling and a healthy cerebrovascular state.

Processed electroencephalogram (EEG) is a commonly utilized technology in the operating room, to objectively assess sedation depth during anesthesia. Bispectral index (BIS) monitoring is the most common processed EEG method to assess sedation depth objectively, with the Entropy index monitoring less prevalent. Both of these indices leverage primarily superficial EEG signals from the frontal lobe (17, 18). However, BIS and Entropy Index adoption for routine monitoring in the ICU has been limited. Furthermore, the association between BIS/Entropy metrics and CBF/cerebrovascular response is uncertain. Though recent work from our laboratory suggests there is the presence of individual patient optimal sedation levels in TBI as measured through BIS (16), such findings are still preliminary and exploratory in nature. Thus, if we are to adopt BIS for continuous assessment of sedation depth in critical and neurocritical care, we require clarity regarding any link between its metrics, neurovascular coupling and CBF/cerebrovascular response. As such, the goal of this study was to perform a systematically conducted scoping review of the literature, assessing for any documented association between BIS and CBF/cerebrovascular reactivity, in humans or animal models.

## Materials and Methods

A systematically conducted scoping review of the available literature was conducted using the methodology outlined in the Cochrane Handbook for Systematic Reviewers (19). The data was reported in line with the Preferred Reporting Items for Systematic Reviews and Meta-Analyses (PRISMA) (20). Appendix A of the Supplementary Materials provides the PRISMA checklist. Search strategy and methodology is similar to other scoping reviews published by our group (3, 21, 22).

The review questions and search strategy were decided upon by the supervisor (F.A.Z.) and primary author (L.F.).

### Search Question, Population, and Inclusion and Exclusion Criteria

The question posed for systematic review was: What is the association between objectively measured depth of sedation, as assessed with processed EEG (i.e., BIS), and the CBF/cerebrovascular response? All studies, either prospective or retrospective, of any size were included. We also included both human and animal studies, to be comprehensive in our scoping overview of the literature.

The primary outcome measure was the association between processed EEG measures and CBF or the cerebrovascular responsiveness, as documented by any neuroimaging technique (i.e., magnetic resonance imaging, computed tomography, PET) or continuous CBF/cerebrovascular monitoring (i.e., laser-Doppler flow probe, transcranial Doppler or any other objective means of CBF determination). Similarly, studies evaluating BIS and cerebral autoregulation/cerebrovascular reactivity, in response to sedation administration, we also included. Secondary outcomes included any other associated physiologic responses to BIS that were documented.

All studies whether prospective or retrospective, of all sizes, human subject or animal models, and with the use of processed EEG (i.e., BIS or Entropy Index) with formal documentation of cerebrovascular response/CBF were eligible for inclusion in this review. Exclusion criteria were the following: being non-English, using non-processed EEG (i.e., not BIS or Entropy Index), or lacking documentation of the association between processed EEG metrics and CBF/cerebrovascular response.

### Search Strategy

MEDLINE, BIOSIS, EMBASE, Global Health, SCOPUS, and Cochrane Library from inception to November 2020 were searched using individualized search strategies for each database. The search strategy for MEDLINE can be seen in Appendix B of the Supplementary Materials, with a similar search strategy used for the other databases. Finally, the reference lists of review articles on the cerebrovascular/CBF response to sedation were examined to ensure no references were left out.

### Study Selection

Using 2 reviewers (LF and JD), a 2-step review of all articles returned by our search strategies was performed. First, the reviewers independently screened all titles and abstracts of the returned articles to decide whether they met the inclusion criteria. Second, full text of the chosen articles was assessed to confirm whether they met the inclusion criteria and that the primary outcome of documented association between processed EEG and CBF/cerebrovascular response. Any discrepancies between the 2 reviewers were resolved by a third party (FZ).

### Data Collection

Data was extracted from the selected articles and stored in multiple electronic databases to ensure data integrity.

### Human Studies

Data fields included the following: number of patients, study type, mean age, patient characteristics, goal of the study, sedation dose and duration, technique to measure CBF/cerebrovascular assessment, CBF/cerebrovascular response, other outcomes and conclusion (i.e., regarding association between BIS and CBF/cerebrovascular response).

### Animal Studies

Data fields included the following: type of models and model characteristics, goal of the study, sedation dose, technique to measure CBF/cerebrovascular assessment, CBF/cerebrovascular response, other outcomes, and conclusion (i.e., regarding association between BIS and CBF/cerebrovascular response).

### Bias Assessment

Given the goal of this review was to provide a comprehensive scoping overview of the available literature, a formal bias assessment was not conducted.

### Statistical Analysis

A meta-analysis was not performed in this study because of the heterogeneity of study designs and data.

## Results

### Search Results and Study Characteristics

**Figure 1:**
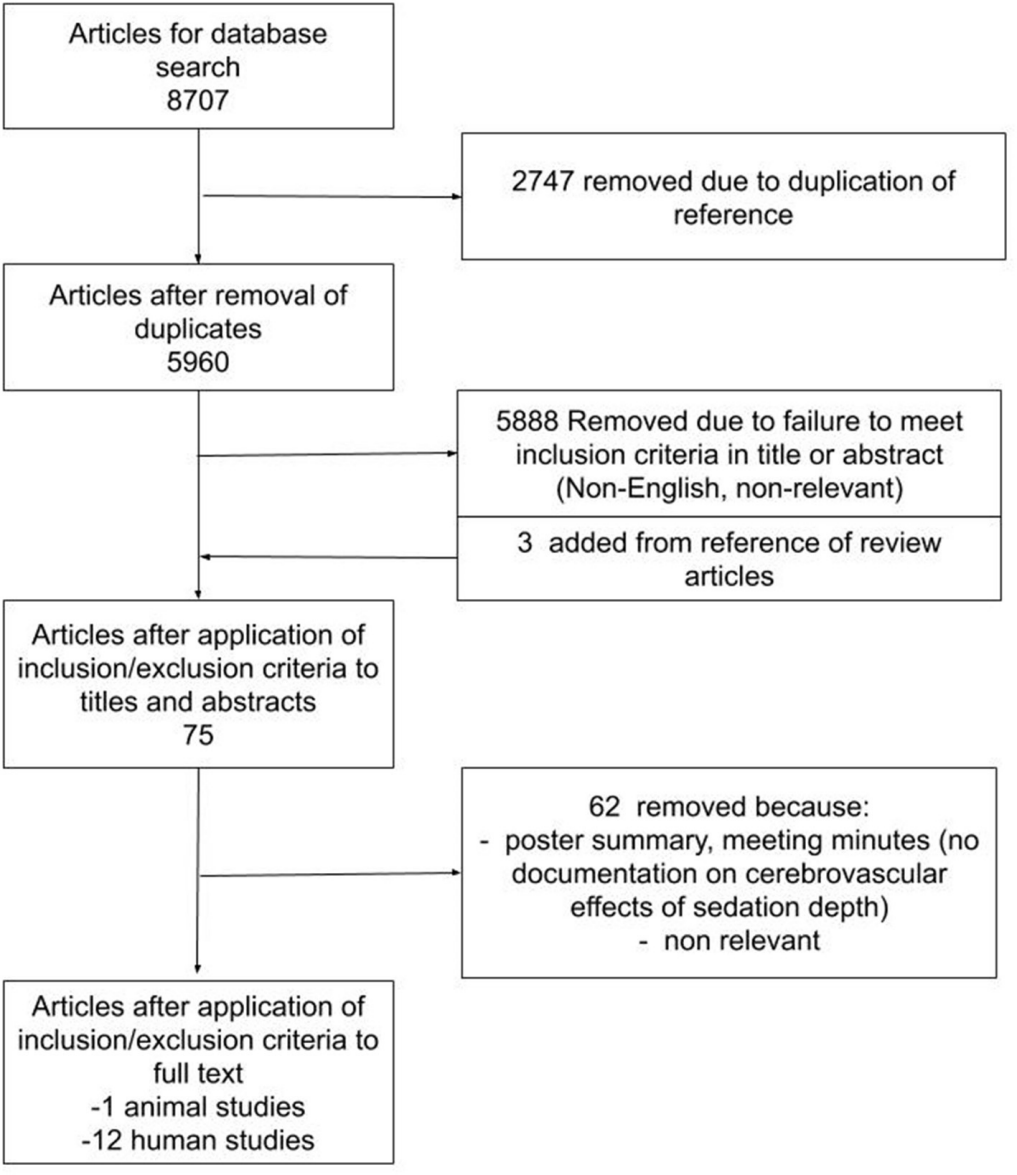
PRISMA Flow Diagram. PRISMA, Preferred Reporting In Systematic Reviews and Meta-Analysis.

The results of the search strategy across all databases and other sources are summarized in Figure 1. Overall, a total of 8,707 articles were identified from the databases searched. A total of 2,747 articles were removed because of duplicated references, leaving 5,960 to review. By applying the inclusion/exclusion criteria to the title and abstract of these articles, we identified 72 articles that fit these criteria. Three articles were added from reference sections of pertinent review articles, leaving a total of 75 full papers to review. On applying the inclusion/exclusion criteria to the full-text documents, only 13 articles were found eligible for inclusion in the systematic review. Articles were excluded because they either did not report details around the association between processed EEG and CBF/cerebrovascular response, were review articles, or were non-relevant. Twelve articles described human adult patients, and the other 1 used animal models. All were original studies, with none describing patients under the age of 18.

Tables 1, 2 show the 12 articles that had human patients and documented the association between processed EEG and CBF/cerebrovascular response (23–34). All articles used the BIS methodology, except for one which used the Entropy Index (23). Patients were either under deep sedation or varying levels of sedation (23, 26, 27), or sleeping (in one study) (24). In order to characterize CBF and vasculature response, the following techniques were used: Positron Emission Tomography (PET) (23, 24, 26–28), transcranial Doppler (25, 31–34) and laser Doppler flowmetry (29, 30). In the human studies, the following cohorts were studied: 7 studies used healthy patients (23–28, 34), two used patients undergoing a craniotomy (29, 30), one used TBI patients (31), one used patients with spinal or maxillofacial disorders (32), and one used patients undergoing carotid endarterectomy (33). The majority of these studies controlled partial carbon dioxide pressure (P_CO2_) through mechanical ventilation (29, 30). Appendix C shows the study that used deeply sedated animal models with BIS recording (35). The animal model study failed to comment on P_CO2_ (35).

**Table 1:** Human included studies–general characteristics and study goals.

**References**	**No.** **patients**	**study type**	**Mean age (y)**	**Patient characteristics**	**Primary and secondary goal of study**
**Healthy patients**					
Maksimow et al. (23)	16	Prospective study	20–30	Healthy male volunteers	Primary: Evaluate the correlation between EEG Entropy and rCBF
Noirhomme et al. (24)	6	Prospective study	20–30	Healthy male volunteers	Primary: Assess the correlation between BIS and rCBF
Ogawa et al. (25)	16	Prospective study	20–26	Healthy young males	Primary: Assess the cerebral circulatory effects of flumazenil after midazolam sedation
Schlünzen et al. (26)	9	Prospective study	21–25	Healthy volunteers	Primary: Evaluate the rCBF effect of sevoflurane Secondary: Differences for sub-anesthesia and anesthesia dose
Schlünzen et al. (27)	9	Prospective study	Not mentioned	Healthy volunteers	Primary: Evaluate the dose-dependent effects of isoflurane on CBF
Veselis et.al. (28)	10	Prospective study	35 ± 10	Healthy male volunteers	Primary: Assess the effects of thiopental and propofol on regions of the brain
**Craniectomy**					
Klein et al. (29, 30)	20	Prospective study	Not mentioned	Patients undergoing a craniotomy	Primary: Assess the effect sevoflurane induced BIS reduction on cerebral microcirculation
Klein et al. (29, 30)	21	Prospective study	35–61	Patients undergoing a craniotomy	Primary: Effect of cerebral microcirculation during propofol infusion
**Head injury**					
Skytioti et al. (31)	17	Prospective study	23–76	ASA physical status of I or II	Primary: ICA flow response to anesthesia, pneumoperitoneum and head-up tilt
**Other adult surgery populations**					
Conti et al. (32)	40	Prospective study	18–65	Patients undergoing treatment for spinal or maxillofacial disorders	Primary: Effect of cerebral hemodynamics after propofol-remifentanil or sevoflurane infusions
Dahaba et al. (33)	20	Prospective study	62.2 ± 9.7	Patients undergoing carotid Endarterectomy	Primary: Detection of CBFv using BIS
Ludbrook et al. (34)	7	Prospective study	18–50	Healthy subjects undergoing elective orthopedic surgery	Primary: Evaluate the effects of Propofol on the Brain

*ASA, American society of anesthesiologist; BIS, bispectral index; CBF, cerebral blood flow; CBFv, cerebral blood flow velocity; CPP, cerebral perfusion pressure; EEG, electroencephalogram; ICA, internal carotid artery; rCBF, regional cerebral blood flow; TBI, traumatic brain injury*.

**Table 2:** Human treatment and cerebrovascular response–study details.

**References**	**Dose**	**Mean duration of dose administration**	**Technique to measure cerebrovascular response**	**Cerebrovascular response**	**Other outcome**	**Conclusions**
**Healthy patients**						
Maksimow et al. (23)	• Sevoflurane: 0.4, 0.7, and 2% • Propofol: 7.6, 12.5 and 19 ug/ml	Not mentioned	• rCBF: PET • Depth of Sedation: EEG Entropy Index	• Both drugs initially decreased BIS and rCBF though after the initial decrease (~5 ml/100 g/min) there was a wide range in BIS and rCBF response • Heavy sedation indicated by BIS did correlate with the lowest rCBF values • P_CO2_ maintained through ventilation	Cortical areas of the most significant associations were remarkably similar for the two drugs	Despite the EEG and rCBF correlation at the extreme end of the spectrum there is a vast amount of internal variations
Noirhomme et al. (24)	Sleep stages		• rCBF: PET • Depth of Sedation: BIS	Linear correlation with rCBF and BIS of up to 0.57 were found at various sleep stages, however BIS values varied widely in both sleep stages and CBF levels		Though the level of rCBF and BIS correlated, there was massive variance within BIS response to sleep stages
Ogawa et al. (25)	• Midazolam: 0.5 mg every 2 min until OAA of 3 • After which flumazenil was administered at 0.2 mg until a OAA of 5	2 H	• CBFv: Transcranial Doppler • ETCO_2_: Pulse oximeter • Depth of Sedation: BIS 4 channel	• For both sedation, BIS and CBFv decreased from baseline values (68 to 64 ± 13 cm/s) with limited change to ETCO_2_ • Despite the increase in BIS level after Flumazenil infusion CBFv still decreased both alone and after midazolam to 61 ± 11 cm/s	Flumazenil reversed the BIS drop of Midazolam without effecting CBFv	Despite the fluctuation in BIS, CBFv remained reduced after sedation, this indicates limited correlation between these values
Schlünzen et al. (26)	Sevoflurane at 0.4, 0.7, and 2%	Not mentioned	• rCBF: PET • Depth of Sedation: BIS	• Sevoflurane decreased the BIS values dose dependently from (96.8 to 38.5 ± 5) • No significant change in global CBF was observed • rCBF increased in the anterior cingulate (17–21%) and decreased in the cerebellum (18–35%), this was identified at all three levels of sedation compared to baseline • P_CO2_ maintained through ventilation		At sevoflurane concentrations at 0.7% and 2.0% a significant decrease in rCBF with dose-dependent decreases to BIS
Schlünzen et al. (27)	Isoflurane: 0.2, 0.4, and 1 MAC	Not mentioned	• rCBF: PET • Depth of Sedation: BIS	• Dose-dependent decrease to BIS (from 96 to 34 ± 6) with Isoflurane infusion but no significant change to global CBF seen • rCBF increased in anterior cingulate and decreased in the cerebellum • P_CO2_ maintained through ventilation		Little correlation with BIS and global CBF
Veselis et.al. (28)	• Propofol: 1.2–2.7 ug/ml • Thiopental: 4.8–10.6 ug/ml	2 H	• rCBF: SPM 99 analysis of PET • Depth of Sedation: BIS • Oxygenation: pulse oximeter	• BIS decreased similar in both sedations, however limited change to rCBF • P_CO2_ maintained through ventilation	Hypnosis drastically reduced BIS level to 70	There is no clear correlation between CBF and BIS
**Craniectomy**						
Klein et al. (29, 30)	Sevoflurane 1.5–2.5% vol/vol	Not mentioned	• Capillary venous blood flow and rSO_2_: Laser-Doppler flowmetry and spectroscopy • Depth of Sedation: BIS	Limited fluctuation from BIS levels of 50–25 had little change to rCBF or rSO_2_		Cerebral microcirculation and oxygenation remains unaltered by sevoflurane-induced changes in BIS
Klein et al. (29, 30)	• Propofol: 4–10 mg/kg/h • Remifentanil: 0.1–0.4 μg/kg/min	Not mentioned	• Capillary venous blood flow and rSO_2_: Laser-Doppler flowmetry and spectroscopy • Depth of Sedation: BIS	• The reduction of BIS from 40 to 21 in both groups had limited results to capillary venous blood flow but propofol had a 20% increase in rSO_2_		Changes in BIS do not seem to influence regional capillary blood flow
**Head injury**						
Skytioti et al. (31)	Propofol: 5.8 to 7.9 mg/kg/h	Not mentioned	• CBFv: Transcranial Doppler Ultrasound • Depth of Sedation: BIS • ETCO_2_: Breath samples • MAP: Finapres	• CBFv and BIS decreased with the introduction of propofol (~100 ml/min) and remained low in both after pneumoperitoneum and head up tilt at 200 ml/min • P_CO2_ maintained through ventilation		Limited correlation from BIS to CBFv as the true measured EEG effects were not commented on
**Other adult surgery populations**						
Conti et al. (32)	Sevoflurane and propofol-remifentanil injected to induce BIS values of 50 and 35	Not mentioned	• CBFv: Transcranial Doppler • Depth of Sedation: BIS • THRR: Calculated from blood flow	• At BIS level of 50 both drugs decreased CBFv (over −10 cm/s) however at BIS 35 sevoflurane saw a slight increase, though this was still less then awake (*p* < 0.05) • Sevoflurane BIS value of 35 had a decrease in THRR to 1.1, propofol-remifentanil had a slight increase to 1.3 baseline was 1.2 • P_CO2_ maintained through ventilation	BIS at level 35 demonstrated similar response as hypercapnia	Propofol–remifentanil demonstrated preservation pressure-flow relationship by inducing a dose-dependent low-flow stateSevoflurane had differing effect on cerebral autoregulation at different concentrationsDespite the BIS and CBFv coupling it is still unclear if this decrease in CBFv is associated with EEG or rather the drug's influence on cerebral circulation
Dahaba et al. (33)	• Propofol: 4 ± 0.2 μg/ml • Rocuronium: 600 ug/kg • Phenylephrine: 50 ug	Not mentioned	• CBFv: Transcranial doppler • Depth of Sedation: BIS • MAP: Controlled with vasopressin	• There was a correlation between BIS and CBFv with a higher correlation after cross clamping of the carotid artery on either side (*p* = 0.112) • Good correlation (r=0.763) between ipsilateral BIS-Vista and CBFv • BIS-Vista decline with CBFv decline both 40% • P_CO2_ maintained through ventilation		• BIS and CBFv had a measurable correlation responsive to lateral influence on the blood flow • BIS-Vista had a discriminative power of depicting a CBFv decline however it cannot be considered a reliable indicator of cerebral ischemia/hypoperfusion.
Ludbrook et al. (34)	Propofol: 110 mg/min for 5 min then 10 mg/min for 20 min	25 Min	• MAP and blood samples: Arterial catheter • CBFv: Transcranial Doppler • Depth of sedation: BIS	• Propofol rapidly dropped BIS after 6.5 min which correlated with low CBFv at 60% of baseline however BIS did carry heavy patient variation • P_CO2_ maintained through ventilation	MAP also had a significant drop at 6.5 min	Propofol and BIS had close relationship together but limited correlation to CBFv

*BIS, bispectral index; CA, cerebral autoregulation; CBF, cerebral blood flow; CBFv, cerebral blood flow velocity; CMRO_2_, cerebral metabolic rate of oxygen; EEG, electroencephalogram; ETCO_2_, end tidal carbon dioxide; MAP, mean arterial pressure; N_2_O, nitrous oxide; P_CO2_, partial pressure of carbon dioxide; PET, positron emission tomography; rCBF, regional cerebral blood flow; rSO_2_, regional oxygen saturation; THRR, transient hyperemic response ratio*.

### BIS Human CBF Response

Overall there was limited direct correlation with BIS and CBF or cerebral blood flow velocity (CBFv), with many studies either demonstrating no correlation (26, 27, 29, 30) or a wide variation in response (24, 25, 28, 34). One study demonstrated BIS having a linear correlation with CBFv and propofol (34), though the variation in CBFv response was significant. Furthermore, *in situations* where consciousness was measured throughout the awake and sedated states, there was a consistent initial decrease in both BIS and CBF/CBFv from the conscious to unconscious state, with sevoflurane (26), propofol (28, 31, 32), or midazolam (25). However, after this initial drop in BIS and CBF/CBFv, there was a wide variation in CBF/CBFv response to similar BIS levels across the population. Of note, one study used midazolam as the initial agent to induce sedation after which flumazenil was used to reverse the sedative effects of midazolam, this increased BIS but did not change CBFv (25). Many of these studies measured various states of consciousness, as evaluated by different levels of BIS, with all having a wide individual variation in CBF/CBFv response at each level (26, 28, 29, 32, 34).

Within the study that evaluated the relationship between CBF and sleep stages, there was a linear correlation between regional CBF and BIS (24). Finally, in a single study that used cross clamping of the carotid artery to modify blood flow, a strong positive correlation between BIS and CBFv was found (33).

### BIS Correlation With Cerebral Vessels and Regional Responses

Only two studies evaluated the capillary venous blood flow response (the blood flow assessed through the capillary bed of the brain) through the use of a laser Doppler flow and spectroscopy. Both studies found that there was limited connection with BIS and cerebrovascular response (29, 30). Three drugs were used to achieve BIS levels of 50 and 21; sevoflurane (29), propofol (30) and remifentanil (30). With each agent there was varying change in blood flow. However, propofol did have a significant increase in regional oxygen saturation by 20% (30). Sevoflurane at high doses (over 0.7%) caused significant decrease to CBF of cerebellum at over 18%, as measured through PET (26). Similarly, in the cortical areas there was a distinct decrease in CBF with a large dose of sevoflurane (over 0.7%) and propofol (over 12.5 ug/ml) (23).

### Entropy Index Human CBF Response

In the single study that used the Entropy Index to assess depth of sedation, there was a wide variation in CBFv response (23). Within this study there were examples of the Entropy Index having a linear correlation with CBFv, during sevoflurane or propofol-remifentanil infusions, when the patient transitioned from awake to sedated states.

### Animal Models

The single animal study used pigs with systemic arterial hypotension and liver trauma. This study found a slight positive correlation between CBFv and BIS, though this lacked statistical significance (35). Coupled with this, BIS was also linked with cerebral tissue oxygenation as measured through near infrared spectroscopy within these models.

## Discussion

Though the literature lacked consistent significant correlations between processed EEG/depth of sedation and cerebrovascular response/CBF, they are undoubtedly associated. This was depicted in all studies that measured BIS or Entropy Index values and CBF/CBFv response, from a conscious to unconscious state. Such studies found that all sedatives caused a decrease in processed EEG values and CBF/CBFv, while mean arterial pressure (MAP) was maintained (23, 25, 26, 28, 31, 32). Furthermore in the one study that clamped the carotid arteries, they found BIS to be correlated with a decrease in CBFv caused through the clamping (33). Though these are limited connections, it highlights that there exists some correlation between objectively measured sedation depth using processed EEG, neurovascular coupling and CBF.

**Figure 2:**
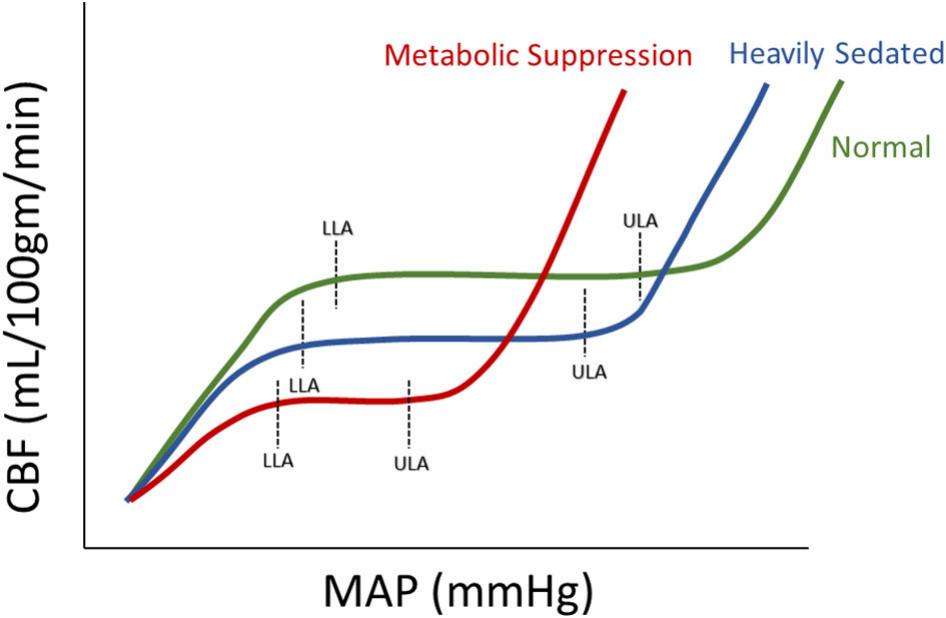
Theoretical Autoregulation curve for Normal, Heavily Sedated, and Metabolically Suppressed Patients. The figure shows three therotical autoregulatory curves for three patient types: normal, heavily sedated and metabolically supressed. a.u., arbitray units; CBF, cerebral blood flow; LLA, lower limit of autoregulation; MAP, mean arterial blood pressure; ml/100 gm/min, milliliter per 100 grams per minute; mmHg, millimeters of mercury; ULA, upper limit of autoregulation.

Within this review, depth of sedation (as measure through BIS or Entropy Index) failed to be clearly linearly associated with CBF. However, this should not be a surprise given CBF is under the control of the innate cerebral autoregulatory function of the pre-capillary arterioles (36). CBF has been shown to follow an S-shaped curve in association with changes in systemic arterial blood pressure, allowing for maintenance of CBF during wide fluctuations of blood pressure. Yet, beyond certain points in MAP, CBF becomes pressure passive. Thus, with escalating sedative doses, corresponding to changes in BIS, there is the potential that we could alter the autoregulatory curve for a given patient, leading to a non-linear relationship between CBF and sedation depth. The influence of sedation on cerebral autoregulation has been demonstrated in past studies (37, 38), and we have illustrated some theoretical responses to sedation in Figure 2. In general, with the introduction of sedation, one would expect lower overall MAP and CBF levels, this would in turn indicate that the plateau of the Lassen curve would be lower than an awake patient. Furthermore, the lower limit of autoregulation would be reduced (emerge at a lower MAP) due to a less exhausted vasodilatory reserve caused through a decrease in metabolic demand (39), with the upper limit of autoregulation being more susceptible to metabolically demanding changes in MAP. There are presumed instances of metabolic suppression where the Lassen curve is greatly deteriorated and thus the lower and upper limits of autoregulation are significantly deranged (40). In such cases, the plateau wave would be greatly reduced or even absent.

**Figure 3:**
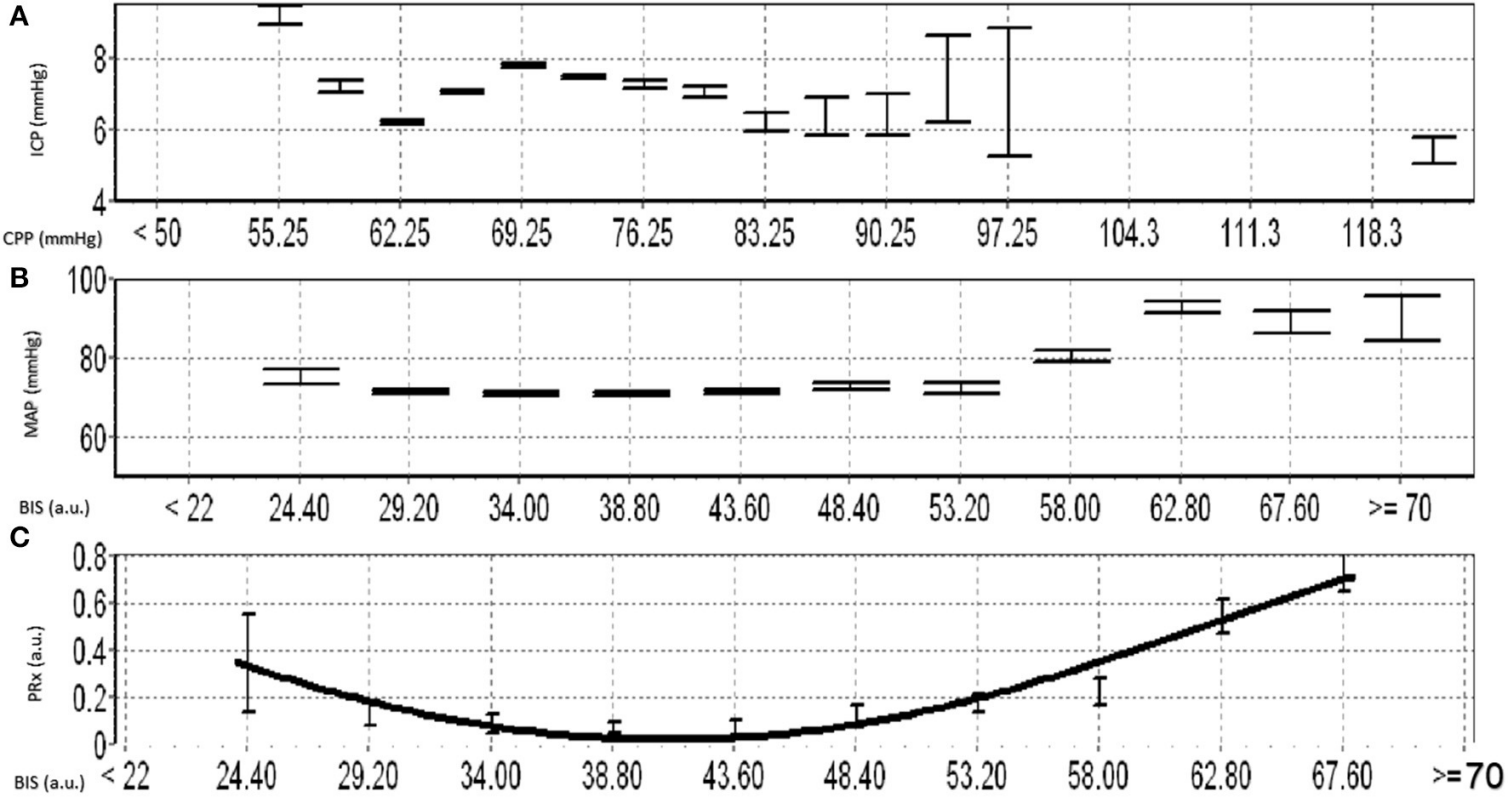
Example of Optimal Depth of Sedation Based on BIS and PRx. **(A)** shows the error bar plot for ICP vs. CPP, **(B)** shows the error bar plot of MAP vs. BIS, **(C)** shows the error bar plot of PRx and different BIS values and demostrates the parabolic relationship between BIS and PRx. a.u., arbitray units; BIS, bisprectal index; CPP, cerebral perfusion pressure; ICP, intracraintal pressure; MAP, mean arterial pressure; mmHg, millimeters of mercury; PRx, pressure reactivity index.

This concept is further supported by recent work from our group, that continuously assessed BIS and cerebrovascular reactivity (using the pressure reactivity index) in high-frequency in TBI patients (16). This exploratory work found that there is a parabolic distribution between BIS and cerebrovascular reactivity, which is patient specific (see Figure 3 for example). We were able to demonstrate deterioration in cerebrovascular reactivity during both light sedation and heavy sedation (i.e., near burst suppression levels) states. Further, these findings in theory could lead to targeted sedation to optimize cerebral autoregulation and reduce secondary insult (16). This is in corollary to such advances seen with individualized optimal cerebral perfusion pressure physiologic targets using cerebrovascular reactivity (41, 42). In this way BIS could be coupled with other forms of cerebral autoregulatory treatment methods to achieve cerebral homeostasis, thus highlighting the impact that processed EEG metric may play in TBI. Furthermore, aside from TBI care, such optimized sedation targets in other critical illnesses may lead to improved cognitive outcomes in general critical care populations though this has yet to be explored.

However, the relationship between objective depth of sedation and CBF or cerebrovascular reactivity is not that simple. This review highlights a vast heterogeneity within the sedative agent used and, as the previous discrepancy of the literate illustrates, each agent may play a different role in cerebral response (3, 4). Thus the limited BIS and CBF connection demonstrated in this heterogeneous body of literature, could be related to disparities in medication type utilized. This was affirmed by the different cerebral responses seen in studies that used two sedative agents to achieve similar BIS values (23, 28, 30). Therefore, the full extent that each sedative agent has on BIS, neurovascular coupling, and CBF/cerebrovascular reactivity is still largely unknown, requiring further investigation.

### Limitations

First, the literature uncovered was very heterogeneous in design, and results had a limited cross-sectional relationship based on the variation of sedative agent used. Second, most studies focused on small patient populations, with limited ability to extrapolate findings. Third, different CBF and cerebral vessel response methods were utilized, which further limits the ability to compare between studies, populations and extrapolate beyond the works identified in this review. Fourth, the disparity in response seen in CBF to changes in processed EEG metrics limits our ability to confidently state the correlation between processed EEG and CBF, highlighting the need for further investigation in this area. Fifth, most studies focused on low-resolution physiology data, in assessing the relationship between processed EEG and CBF/cerebrovascular reactivity. Such data is limited in its ability to explore the temporal profile of objective sedation depth changes, using BIS, and CBF/cerebrovascular response. This highlights the need for continuous high-fidelity data sets, with BIS and multi-modal cerebral physiologic monitoring to properly comment on any associations.

### Future Directions

Despite the identified limitations of our review and the knowledge gap in the literature, there are essential avenues for future investigation highlighted by this work. First, metrics that focus on processed EEG like BIS or Entropy Index, use targeted algorithms to reduce the highly variable and vastly complex EEG output of the superficial area of the frontal lobe. Thus, if these metrics are implemented to help evaluate CBF or autoregulation the clinician must be aware that any change to the normal electrical impedance of the fontal cortex or the areas of interest are elsewhere, the results would have impeded accuracy. Therefore, studies will require high-frequency data streams of processed EEG metrics, linked with multi-modal cerebral physiologic monitoring to expose the more consistent physiological response and reduce confounding factors. Spatial resolution on EEG entropy index assessments could be improved with large EEG arrays, with signals process for each channel. Similarly, as PRx is derived from a focal pressure monitoring, future improvements in spatial resolution for autoregulation assessments may be facilitated by multi-channel functional near infrared spectroscopy or bilateral transcranial Doppler assessments.

The analysis of these continuous variables in conjunction with processed EEG, will allow the researcher to comment on the multiple factors that influence BIS like MAP, severe cerebral ischemia, impaired autoregulation and P_CO2_. Along with this, devices like near infrared spectroscopy or parenchymal brain tissue oxygen probes would both potentially offer both the assessment of regional cerebral oxygen delivery, in concert with sedation depth and cerebral autoregulation.

Additionally, multi-modal cerebral physiologic data linked with medication dosing information in time-series would also aid in the understanding of various sedative agents and their subsequent impact on physiology and BIS. As well, by pairing the dosing regimen the researcher can account for the influence of other confounding factors in these agents like MAP or the metabolic coupling effect. Furthermore, targeted sedation strategies using propofol or barbiturates that have similar effects globally throughout the brain (3, 43) would better isolate discrepancy between BIS response and outside confounding factors.

Finally, when assessing the parabolic relationship between BIS and PRx, the use of time connected high frequency physiological data would provide better insight as to the true impairment of the Lassen curve and optimal BIS values. Current literature assessing cerebral autoregulation and metabolic suppression is limited, and is hampered by global assumption about BIS response and sedation. Factors like subdural hemorrhage causing fluctuations to regional electrical impedance, ischemia/systemic vasopressors/blood gas levels causing metabolic fluctuations or other systemic stimuli triggering increase brain activity, all result in derangements to BIS values. Thus, continuous data sets would allow the analysis of separate physiological responses and patient states throughout treatment. Opening the opportunity to comment on the interconnected nature of processed EEG to other cerebral states.

All of this information will need large multi-center data sets with, studying a variety of critical illness states, healthy patients undergoing elective surgery, and awake volunteers. Such comprehensive data collection strategies will highlight the relationships between sedation depth and cerebrovascular response. The findings here will better delineate the role of processed EEG in routine monitoring for patients with critical illness and potentially the role of individualized sedation metrics to advance personalized medicine approaches in critical care (16). Such work is the focus of our lab, the Winnipeg Acute TBI Laboratories, and various research collaboratives (16, 44–47).

## Conclusions

This review highlights the potential for processed EEG metrics to provide information regarding CBF/cerebrovascular response. The literature demonstrates that initiation of sedation will decrease BIS/Entropy Index, CBF and CBFv, highlighting processed EEG's potential to quantify neurovascular coupling. However, after this initial decrease there is a wide range of response between BIS and CBF/CBFv seen, both within and between patient cohorts. Thus, any conclusion about sedation and its role on neurovascular coupling and cerebrovascular response is uncertain. Variation in responses may be related to the differential effects of sedative agents on individual subject's autoregulatory function and/or patient's depth of sedation. Future research with high frequency datasets is required to evaluate processed EEG/BIS and its correlation with CBF/cerebral autoregulation.

## Author Contributions

LF was responsible for design, analysis and manuscript composition. JD was responsible for article screening and manuscript composition. AG, CB, and AS were responsible for manuscript composition. FZ was responsible for concept, design, analysis, manuscript composition, and supervision. All authors contributed to the article and approved the submitted version.

## Conflict of Interest

The authors declare that the research was conducted in the absence of any commercial or financial relationships that could be construed as a potential conflict of interest.

## Publisher's Note

All claims expressed in this article are solely those of the authors and do not necessarily represent those of their affiliated organizations, or those of the publisher, the editors and the reviewers. Any product that may be evaluated in this article, or claim that may be made by its manufacturer, is not guaranteed or endorsed by the publisher.
