# Supplementary material for: Association Between Processed Electroencephalogram-Based Objectively Measured Depth of Sedation and Cerebrovascular Response: A Systematic Scoping Overview of the Human and Animal Literature
Source: Front Neurol. 2021 Aug 16;12:692207. doi: 10.3389/fneur.2021.692207 (PMC8415224; doi:10.3389/fneur.2021.692207)
Supplement: Supplementary file 2 [file Data_Sheet_2.doc]

Appendix B. Ovid search

1 Cerebrovascular Circulation.mp. [mp=ti, ab, hw, tn, ot, dm, mf, dv, kw, fx, dq, bt, id, cc, nm, kf, ox, px, rx, ui, sy]

2 Cerebral Blood Flow.mp. [mp=ti, ab, hw, tn, ot, dm, mf, dv, kw, fx, dq, bt, id, cc, nm, kf, ox, px, rx, ui, sy]

3 Cerebral Circulation.mp. [mp=ti, ab, hw, tn, ot, dm, mf, dv, kw, fx, dq, bt, id, cc, nm, kf, ox, px, rx, ui, sy]

4 Cerebral Perfusion Pressure.mp. [mp=ti, ab, hw, tn, ot, dm, mf, dv, kw, fx, dq, bt, id, cc, nm, kf, ox, px, rx, ui, sy]

5 Circulation, Cerebrovascular.mp. [mp=ti, ab, hw, tn, ot, dm, mf, dv, kw, fx, dq, bt, id, cc, nm, kf, ox, px, rx, ui, sy]

6 CBF.mp. [mp=ti, ab, hw, tn, ot, dm, mf, dv, kw, fx, dq, bt, id, cc, nm, kf, ox, px, rx, ui, sy]

7 cbfv.mp. [mp=ti, ab, hw, tn, ot, dm, mf, dv, kw, fx, dq, bt, id, cc, nm, kf, ox, px, rx, ui, sy]

8 cpp.mp. [mp=ti, ab, hw, tn, ot, dm, mf, dv, kw, fx, dq, bt, id, cc, nm, kf, ox, px, rx, ui, sy]

9 Cerebral Homeostasis.mp. [mp=ti, ab, hw, tn, ot, dm, mf, dv, kw, fx, dq, bt, id, cc, nm, kf, ox, px, rx, ui, sy]

10 cerebral auto regulation.mp. [mp=ti, ab, hw, tn, ot, dm, mf, dv, kw, fx, dq, bt, id, cc, nm, kf, ox, px, rx, ui, sy]

11 Intracranial Pressure.mp. [mp=ti, ab, hw, tn, ot, dm, mf, dv, kw, fx, dq, bt, id, cc, nm, kf, ox, px, rx, ui, sy]

12 Intracerebral Pressure.mp. [mp=ti, ab, hw, tn, ot, dm, mf, dv, kw, fx, dq, bt, id, cc, nm, kf, ox, px, rx, ui, sy]

13 Subarachnoid Pressure.mp. [mp=ti, ab, hw, tn, ot, dm, mf, dv, kw, fx, dq, bt, id, cc, nm, kf, ox, px, rx, ui, sy]

14 ICP.mp. [mp=ti, ab, hw, tn, ot, dm, mf, dv, kw, fx, dq, bt, id, cc, nm, kf, ox, px, rx, ui, sy]

15 Cerebral blood flow velocity.mp. [mp=ti, ab, hw, tn, ot, dm, mf, dv, kw, fx, dq, bt, id, cc, nm, kf, ox, px, rx, ui, sy]

16 cerebrovascular reactivity.mp. [mp=ti, ab, hw, tn, ot, dm, mf, dv, kw, fx, dq, bt, id, cc, nm, kf, ox, px, rx, an, ui, sy]

17 1 or 2 or 3 or 4 or 5 or 6 or 7 or 8 or 9 or 10 or 11 or 12 or 13 or 14 or 15 or 16

18 EEG.mp. [mp=ti, ab, hw, tn, ot, dm, mf, dv, kw, fx, dq, bt, id, cc, nm, kf, ox, px, rx, an, ui, sy]

19 processed EEG.mp. [mp=ti, ab, hw, tn, ot, dm, mf, dv, kw, fx, dq, bt, id, cc, nm, kf, ox, px, rx, an, ui, sy]

20 depth of sedation.mp. [mp=ti, ab, hw, tn, ot, dm, mf, dv, kw, fx, dq, bt, id, cc, nm, kf, ox, px, rx, an, ui, sy]

21 Consciousness Monitor.mp. [mp=ti, ab, hw, tn, ot, dm, mf, dv, kw, fx, dq, bt, id, cc, nm, kf, ox, px, rx, an, ui, sy]

22 Index of consciousness-View.mp. [mp=ti, ab, hw, tn, ot, dm, mf, dv, kw, fx, dq, bt, id, cc, nm, kf, ox, px, rx, an, ui, sy]

23 Index of Consciousness View Monitor.mp. [mp=ti, ab, hw, tn, ot, dm, mf, dv, kw, fx, dq, bt, id, cc, nm, kf, ox, px, rx, an, ui, sy]

24 Bispectral Index Monitor.mp. [mp=ti, ab, hw, tn, ot, dm, mf, dv, kw, fx, dq, bt, id, cc, nm, kf, ox, px, rx, an, ui, sy]

25 Bispectral Index.mp. [mp=ti, ab, hw, tn, ot, dm, mf, dv, kw, fx, dq, bt, id, cc, nm, kf, ox, px, rx, an, ui, sy]

26 Cerebral State.mp. [mp=ti, ab, hw, tn, ot, dm, mf, dv, kw, fx, dq, bt, id, cc, nm, kf, ox, px, rx, an, ui, sy]

27 Electroencephalogram.mp. [mp=ti, ab, hw, tn, ot, dm, mf, dv, kw, fx, dq, bt, id, cc, nm, kf, ox, px, rx, an, ui, sy]

28 Electrodiagnosis.mp. [mp=ti, ab, hw, tn, ot, dm, mf, dv, kw, fx, dq, bt, id, cc, nm, kf, ox, px, rx, an, ui, sy]

29 18 or 19 or 20 or 21 or 22 or 23 or 24 or 25 or 26 or 27 or 28

30 17 and 29

31 1 or 2 or 3 or 4 or 5 or 6

32 7 or 8 or 9 or 10 or 11 or 12 or 13 or 14 or 15 or 16

33 29 and 31

34 29 and 32

35 remove duplicates from 33

36 34 or 35

37 remove duplicates from 36
