# Supplementary material for: Association Between Processed Electroencephalogram-Based Objectively Measured Depth of Sedation and Cerebrovascular Response: A Systematic Scoping Overview of the Human and Animal Literature
Source: Front Neurol. 2021 Aug 16;12:692207. doi: 10.3389/fneur.2021.692207 (PMC8415224; doi:10.3389/fneur.2021.692207)
Supplement: Supplementary file 3 [file Data_Sheet_3.docx]

Appendix C. Animal Study

| **References** | **Patient Characteristics** | **Dose** | **Technique to Measure Cerebrovascular Response** | **Cerebrovascular Response** | **Primary and Secondary Goal of Study** | **Conclusions** |
| --- | --- | --- | --- | --- | --- | --- |
| Cavus et al^33^ | 16 pigs with severe hypotension and liver trauma | Deep sedation: given ketamine (20 mg/kg) propofol (1-2 mg/kg) and sufentanil (0.2 ug/kg) then maintained with propofol and sufentanil | Tissue oxygenation: NIRS  CBFv: Transcranial Doppler  One channel BIS | BIS was positively correlated with tissue oxygenation and CBFv however this lack statistical significance  BIS was neutral with propofol plasma concentration | Primary: Effects of cerebral hypoperfusion on BIS | BIS was not a strong indication of CBFv and tissue oxygenation, with a confusing lack of correlation with propofol concentration |

BIS, bispectral index; CBF, cerebral blood flow; CBFv, cerebral blood flow velocity; CMRO_2,_ cerebral metabolic rate of oxygen; EEG, electroencephalogram; H_2_, Dihydrogen; MAP, mean arterial pressure; N_2_O, nitrous oxide
